# Effect of Stirring Needle Length on the Microstructures and Properties of A380/6061 Dissimilar Aluminium Alloy FSW Joints

**DOI:** 10.3390/ma18071621

**Published:** 2025-04-02

**Authors:** Xinbin Hu, Zhongxu Luo, Sheng Liu, Yongjun Ren, Wei Long

**Affiliations:** 1 School of Materials and Chemical Engineering, Hubei University of Technology, Wuhan 430068, China; m18827533817@163.com (Z.L.); r736574180@163.com (Y.R.);; 2 Key Laboratory of Green Materials for Light Industry of Hubei Provincial, Wuhan 430068, China; 3 Hubei Engineering Laboratory of Automotive Lightweight Materials and Processing, Wuhan 430068, China

**Keywords:** aluminium alloy, FSW joints, needle lengths, RS-HAZ zone, grain size

## Abstract

In this study, FSW experiments were conducted on a 4 mm-thick A380 aluminium alloy plate and 6061 aluminium alloy using four different needle lengths (2.5 mm, 2.8 mm, 3.0 mm, and 3.5 mm) of H13 steel. The experiments were conducted at the same welding parameters (1000 rpm and 120 mm/min) to explore the effects of different stirring needle lengths on the microstructures and properties of the FSW joints. The experimental results show that FSW joints with varying lengths of needles have a significant effect on the microstructures and properties of welded joints, and the tensile strength increases and then decreases with increases in the needle length. A 2.8 mm needle length can achieve the maximum tensile strength of 203 MPa, about 85.3% of the base material. Too long or too short a needle leads to a decrease in joint performance. Furthermore, different needle lengths have a significant influence on the flowability of the weld core zone, and a suitable needle length will lead to better flowability in the weld core zone. With increases in the needle length, the heat production also increases, and the area of the RS-HAZ will increase with the heat production. When the joints achieve appropriate heat production, the weld core zone will experience grain refinement. At the same time, the grain will grow in the RS-HAZ; the hardness cloud diagram shows that, in the RS-HAZ, the material properties are weaker, and the tensile specimens are mainly fractured in the RS-HAZ. Finally, the tensile specimens exhibit mixed fracture.

## 1. Introduction

The widespread application of lightweight materials has promoted the development of automotive lightweighting. Further, aluminium alloy material offers the advantages of great corrosion resistance, high strength, great electrical conductivity, and high thermal conductivity [1,2], which makes it one of the most widely used lightweight materials. Moreover, 60% of the total energy consumption of a car comes from its weight, and for every 10% reduction in body weight, the energy consumption of the car is reduced by 10–15% [3]. Different aluminium alloys are used in different scenarios; cast aluminium and deformed aluminium are mainly used in wheel hubs, sub-frames, and battery packs. Therefore, cast aluminium alloys and deformed aluminium alloys are becoming more and more widely used in the automotive field [4,5,6,7]. Such applications inevitably involve the joining of two aluminium alloys.

Since aluminium alloys have a large thermal expansion coefficient and thermal conductivity, conventional welding methods such as tungsten-pole argon arc welding [8,9], laser-MIG composite welding [10,11], fusion-pole argon arc welding [12], and laser welding [13] involve excessively high melting temperatures, which makes the welded joints very prone to forming defects such as pores and cracks. This will, in turn, significantly affect the microstructural properties of the welded joints. In particular, the cast aluminium alloy and 6061 aluminium alloy involve differences in properties [14,15,16], which increases the complexity of melt welding. Friction stir welding (FSW) is a new solid connection technology invented by the British Institute of Welding in 1991, which offers significant advantages when welding dissimilar metals. Its low temperature during the welding process does not produce fumes, gases, or impurities, and the base material does not melt, which reduces the generation of brittle intermetallic compounds (IMCs) and realises green manufacturing. The welded joints obtained using this method offer high strength, great sealing, and high corrosion resistance, improving their overall quality.

Research on the stir friction welding of dissimilar aluminium alloys is relatively extensive. To optimise the welding process parameters, Majid F. [17] studied the mechanical strength and microstructure of friction stir-welded joints of AA2024-T6 aluminium alloy; it was found that, with an increase in rotational speed, the grain size in the stirred zone becomes larger, in addition to obtaining a uniform second-phase distribution. By increasing the rotational and transverse speeds, the hardness in the thermomechanically affected zone and stirred zone increased to the base metal hardness. The maximum tensile strength of these welded plates fractured on the advancing side was equal to 71% of the strength of the substrate obtained at a transverse speed of 31.5 mm/min and a rotational speed of 1120 rpm. At a constant speed, the longitudinal residual stress decreases with a decrease in speed of 1120 rpm. Zulai H. [18] studied the effect of (TA) on the forming of stir friction lap welds of 2A12-T42 aluminium alloy. It was found that, when TA increased from 2° to 5°, the shear resistance of the lap joint first increased and then decreased, the maximum shear resistance of 3961.3 N could be obtained when the welding inclination angle was 3°, the welding inclination angle did not have a great influence on the grain size of the SZ zone, and the SZ zone and the TMAZ zone of the welded joints appeared to be partly adhesive at 2°, which led to the deterioration of the fluidity and performance degradation, and the welded joints seemed to have a large HOOK-shaped defect at 5°, which led to the degradation of the performance of the joints. Defects in the welded joints at 5° resulted in a decline in joint performance. Pandav N A. [19] and others explored the optimum process parameters for AA2050 aluminium alloys through orthogonal experiments using L16, which tested the tensile strength, yield strength, elongation, weld zone hardness, bending load, etc. Through Taguchi-GRA-PCA analysis, the most suitable process parameters were determined to be 900 rpm, a welding speed of 160 mm/min, and a tilt angle of 2°. ANOVA revealed the correlation of all the five characteristics; the rotational speed had the greatest influence, accounting for 43.56% of the overall results. Confirmatory experiments carried out under ideal conditions exhibited a significant increase in the total weld quality of 19.06%. Dehabadi V M, Delijaicov S, and Kareem N [20,21,22] predicted the effect of tool rotational speed and welding speed on the mechanical properties, type, and distribution of residual stresses in the joints. The differences in thermal and mechanical properties of the material under different process parameters resulted in a complex distribution of residual stresses; the longitudinal residual stress component was asymmetrically distributed in the direction of the plate width in the shape of the letter ‘M’, and the maximum tensile value of the forward side was greater than that of the backward side. Zhao Zimo et al. [23] found that the fatigue strength of friction-stir-welded joints of dissimilar materials is lower than the strength of lap joints of the same material. The fatigue performance is related to the transformation of the β’phase to the coarsened β’phase, the brittle second phase, the inhomogeneous mixing at the junction of the weld core and thermomechanically affected zone, and the co-superposition of stress concentration. Rasoul K S. [24] concluded that the poor matching of the tool rotation speed and traverse speed leads to an increase in the heat production rate of welded joints. With increasing heat production rates, the number of intermetallic compounds and voids of Al_4_Cu_9_ and Al_2_Cu in the stirred zone increases, resulting in lower strength, lower ductility, and higher resistivity for the welded joints. The residual tensile stress zone near the aluminium side extends along the weld zone and HAZ, and a narrower residual compressive stress zone is formed near the copper side closer to the interface.

To analyse different mixing tool shapes, Wu X et al. [25] fusion-welded 6061 aluminium alloy with QP980 steel using the friction stir welding (FSW) technique, and they investigated the effect of 1.5 mm and 2.1 mm probe lengths on the microstructures and properties of the welded joints. When the probe length was 2.1 mm, scattered steel chips were present in the aluminium layer. Two intermetallic compounds could be detected: the dark grey layer close to the Al was the Fe_4_Al_13_ phase, and the one close to the steel was the Fe_2_Al_5_ phase. The steel debris embedded in the Al matrix promotes stress concentration and crack initiation during deformation, which reduces the mechanical properties of the joints. Arutiun E et al. [26] used the stationary-shoulder friction stir welding (SS-FSW) technique, which benefits from a reduced heat input, improved mechanical properties, and better surface finish for the weld. The addition of nanoscale multilayers of Ti AlN/VN to the tool probe and shoulder improved the efficiency and quality of the weld and reduced tool wear. Ren D et al. [27] proposed friction stir welding with cover (CFSW) to address the thinning caused by the tool shoulder and reduce the heat-affected zone. The microstructure and mechanical properties of CFWS were also investigated. With the addition of a cover, reinforcement is formed on the weld surface to compensate for the thinning caused by friction in the tool shoulder. When the cover absorbed heat from the shoulder, the width of the heat-affected zone of the weld plate became smaller than the diameter of the shoulder. The tensile strength of 5754 aluminium alloy joints reached 94% of the tensile strength of the base material without grinding the cover plate. Verma S et al. [28] evaluated the mechanical and metallurgical properties of the joints by using six different tool pin shapes and correlating them with the grain size in the melting core zone and thermal properties. The swept volume ratio and pulsation stirring action of the pin profiles were also quantified. The square pin profile provided excellent mechanical and metallurgical properties due to higher pulsation stirring action and a sufficient sweep volume ratio.

A380 is a type of Al-Si casting aluminium alloy, which offers the advantages of great plasticity, high hardness, and lightweight properties; 6061 is a type of Al-Mg-Si deformed aluminium alloy, which offers great corrosion resistance, plasticity, and weldability. Although there are a large number of studies on the effect of cast aluminium/deformed aluminium alloy stir friction welding joint structures and properties, there is a lack of understanding regarding the effects of FSW joint parameters. Thus far, there has been no research on the welding process parameters and FSW joint parameters for cast aluminium/deformed aluminium alloy friction stir welding joints.

This paper studies the use of a 4 mm plate thickness of A380 and 6061 for friction-stir welding. The welding method used is docking, and the FSW joint needle length is changed to ascertain its impact on the microstructural properties of the welded joints. Changing the needle length can improve the fluidity of the weld metal and significantly improve the performance of the joints and the joint strength coefficient. The method proposed in this paper can be used to optimise the performance of the joints of the cast aluminium/deformed aluminium alloys and act as theoretical guidance.

## 2. Materials and Methods

### 2.1. Materials

**Figure 1:**
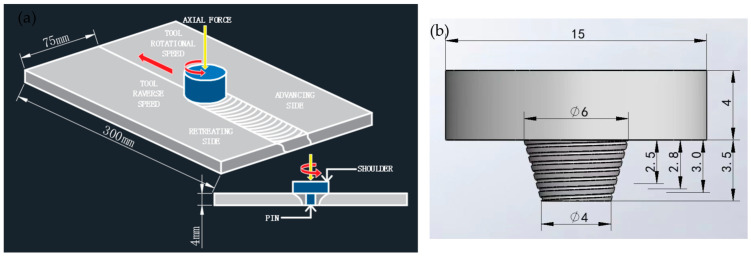
A380/6061 dissimilar aluminium alloy FSW joints in experimental programme: (**a**) schematic of welding; (**b**) schematic of the shape and size of the FSW joints.

Two types of aluminium plates were used for the experiments, namely cast aluminium A380 and 6061-T6. Their dimensions were 300 mm × 75 mm × 4 mm. The specific chemical compositions of the two materials are shown in Table 1. The model FSW-TS-08 (Beijing Safest Technology, Beijing, China) friction stir welding machine was used for welding. Before welding, the surface to be welded and the docking surface were polished with 400-mesh sandpaper to remove the oxidised layer and then rinsed with anhydrous ethanol and prepared for use. The welding method was docking, the 6061-T6 aluminium plate was placed on the forward side, the A380 aluminium plate was placed on the backward side, the spindle rotating speed was 1000 rpm, the rotating speed was 120 mm/min, and the welding inclination angle was 2°; the welding diagram is shown in Figure 1a. The tensile strength of the A380 base material was 238 MPa, and that of the 6061 base material was 256 MPa. The tensile strength dimensions of the base material are in accordance with international standards. The tensile strength dimensions of the parent material are in accordance with international standards. The material of the mixing joint is H13 steel; the shoulder diameter of the mixing joint is 15 mm; the pin lengths are 2.5 mm, 2.8 mm, 3.0 mm, and 3.5 mm, respectively; and the shape is a round table with threads. The specific dimensions are shown in Figure 1b.

**Figure 2:**
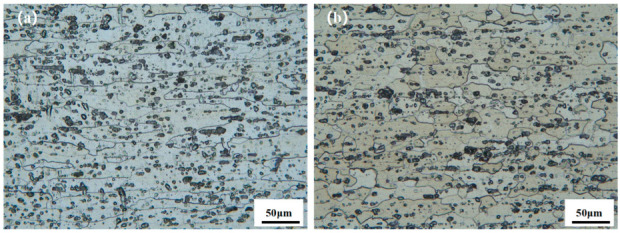
Microstructure of two aluminium alloy base materials: (**a**) A380; (**b**) 6061.

Figure 2 shows the microstructure of A380 and 6061-T6 aluminium alloy base material; Figure 2a is the A380 base material microstructure, where the grain morphology is flat and fibrous, accompanied by black precipitates on the surface. The average size of the grains is 20.1 μm, and the grain size is coarse. Figure 2b shows the microstructure of 6061-T6, where the shape of the grains is not uniform, there are a large number of reinforcing Mg_2_Si phases, and the size of the grain is slightly smaller, at 15.7 μm.

### 2.2. Experimental Procedure

**Figure 3:**
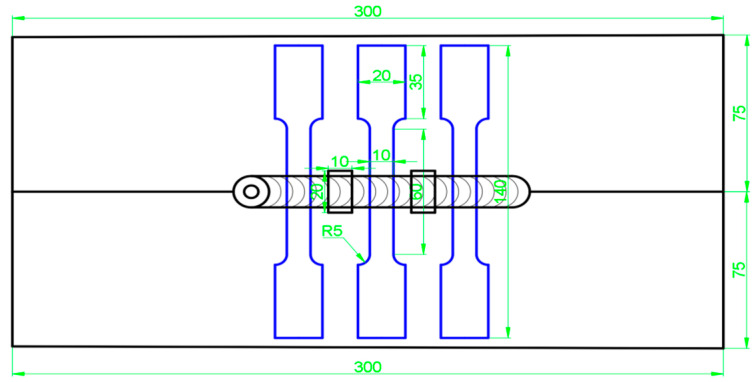
Schematic diagrams of fishbone sampling and metallographic sampling with A380/6061 dissimilar aluminium alloy FSW joints.

After the welding was completed, fishbone and metallographic specimens were cut in the middle area of the weld using an EDM wire cutter. Tensile and metallographic observations were then carried out. The specimens were then cut along the vertical direction of the weld, with the centre of the weld as the benchmark on both sides of the intercept of 10 mm, a specimen size of 20 mm × 10 mm × 4 mm, including the base metal (BM), heat-affected zone (HAZ), thermo-mechanically affected zone (TMAZ), and weld core. The TMAZ and weld nugget zone (WNZ) of the metallographic specimens and fishbone specimens were in accordance with the national standards for the design of these specimens. The sampling schematic of the metallographic specimens and fishbone specimens is shown in Figure 3.

All the specimens were sequentially ground with #400, #600, #800, #1000, #1200, #1500, #2000, and #3000 water sandpaper until the surface became flat and polished. They were then cleaned with anhydrous ethanol, blown dry, and subsequently put into a vacuum drying box. Keller’s reagent (95 mL of pure water + 2.5 mL of HNO_3_ + 1.5 mL of HCl + 1 mL of HF) was used to corrode the welded joints, and the corrosion time was 15–20 s. A GX51 (Chongqing Yongchang Technology, Chongqing, China) metallurgical microscope (Olympus, OM, Tokyo, Japan) and scanning electron microscope (SEM, ZEISS GeminiSEM 300, Oberkochen, Germany) were used to analyse the microstructure of the joints. The microstructure of the weld region was analysed via energy dispersive spectroscopy (EDS); the composition of the physical phase of the joints was analysed via electrolytic polishing. EBSD samples were prepared via electrolytic polishing, and the samples were analysed through electron backscattered diffraction (EBSD) using an Oxford Nordlys max3 (Oxford, UK). EBSD was used to observe the grain boundaries and grain size, with a scanning step of 0.5 um; the Image Pro Plus 6.0 software was used to determine the grain size in the SZ zone, the grain size in the RS-HAZ, and the area size in the RS-HAZ of a typical sample. The hardness values of the metallographic specimens were measured with a Vickers hardness tester (UMT-VMHT VMH-104, München, Germany), and a load of 50 g was applied to the sample for 10 s. The hardness of the entire welded joint cross-section was measured at a horizontal and vertical spacing of 0.5 mm and 0.4 mm, respectively, and the hardness cloud was plotted; each sample was tested 351 times. The K-type single-channel thermocouple was used to collect the temperature of the welding process in real time; the collection time was 200 s, the collection rate was 4 s, and the punched position was about 5 cm from the centre of the weld.

This experiment was carried out to investigate the effect of different needle lengths on the microstructural properties of welded joints by choosing the same spindle speed and welding speed and selecting the same amount of downward pressure as the needle length, with the aim of making the joints unaffected by the shaft shoulder during welding.

## 3. Results and Discussion

### 3.1. Macro-Discussion of Joint Morphology

**Figure 4:**
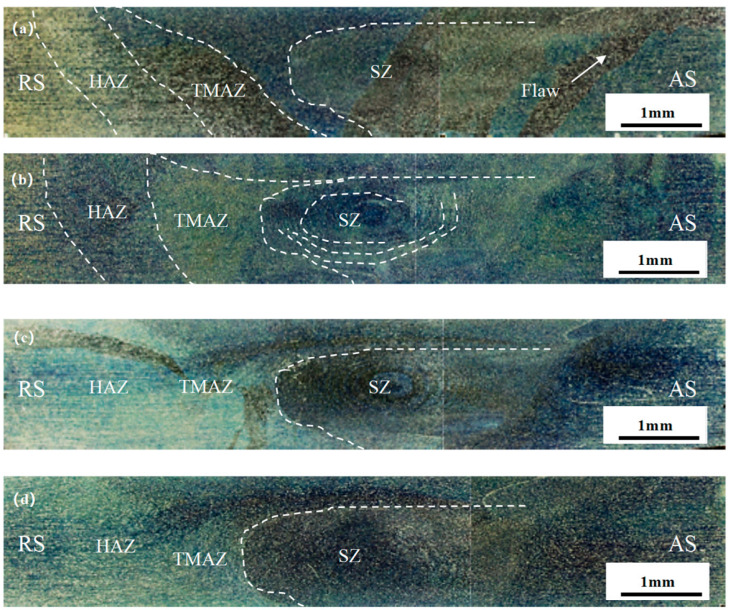
FSW joints of A380/6061 dissimilar aluminium alloys with different stirring pin lengths in macroscopic morphology under a stereomicroscope; (**a**) 2.5 mm; (**b**) 2.8 mm; (**c**) 3.0 mm; and (**d**) 3.5 mm.

Figure 4 shows the welding experiments of stirring heads, with four different needle lengths, at the optimum rotational and welding speeds. There is an obvious area of stratification, and Figure 4a corresponds to the 2.5 mm needle length. There are obvious defects in the TMAZ zone and SZ zone, which is the advancing side of the FSW joints. Further, spatter is formed on the A380 area because the stirring needle length is too short, the heat generated via the weld is not sufficient, and the material in the weld core area does not achieve good mechanical bonding. These defects deteriorate the performance of the welded joints. Figure 4b corresponds to a needle length of 2.8 mm. When the stirring needle is extended to the middle of the weld, the generated heat increases, and the material is mechanically bonded in the weld core area. A clear swirl-like structure appears in the weld core zone, the material flows well, and no band defects are produced in the RS-HAZ. This indicates that the material has performed well. Figure 4c corresponds to a 3.0 mm needle length, where the heat generated via the stirring needle continues to increase, and a clear, swirl-like structure can still be seen in the weld core zone. There is good fluidity, but there are obvious banding defects in the RS-HAZ and TMAZ zones. These defects will deteriorate the performance of the welded joint. Figure 4d corresponds to a 3.5 mm needle length; the material flow in the weld core zone is weak, and there are still some defects in the middle of the SZ zone and the TMAZ zone. These irregular defects deteriorate the performance of the welded joint.

### 3.2. Effect of Welding Temperature

**Figure 5:**
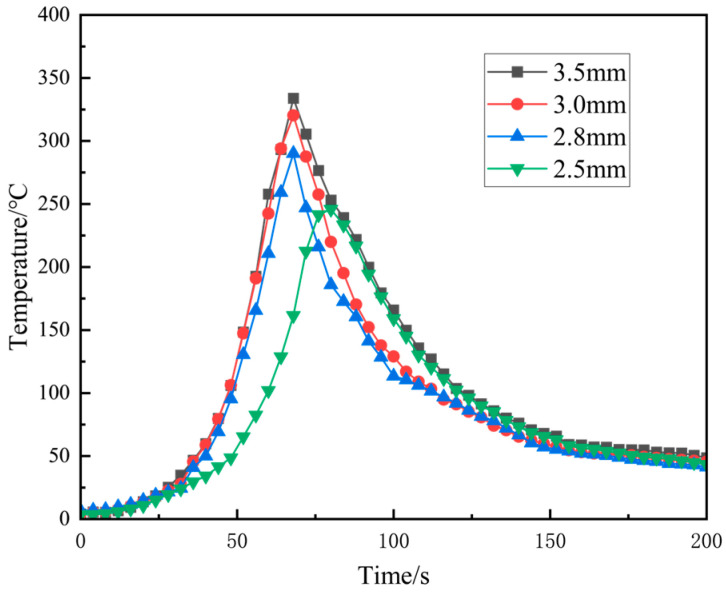
FSW joints with different stirring pin lengths for A380/6061 dissimilar aluminium alloys: welding heat cycle curve.

Figure 5 shows the thermal cycle curves at 5 mm from the centre of the weld for different needle lengths, where a large amount of heat is generated due to the direct friction between the FSW joint and the welded material. When the position of the weldment reaches near the temperature measurement point, the temperature rises sharply, and when the weldment moves away from the temperature measurement point, the temperature starts to decrease slowly to room temperature. It can be seen from Figure 5 that, as the needle length of the stirring needle increases, the maximum temperature generated also increases. A needle length of 3.5 mm can reach a maximum temperature of 333.8 °C, 3 mm can reach a maximum temperature of 320 °C, 2.8 mm can reach a maximum temperature of 290.1 °C, and 2.5 mm can reach a maximum temperature of 245.8 °C. This trend coincides with the frictional heat generation formula of the FSW joint and the material:

(1) $$Q=\frac{4\Pi^{2}}{3}\mu NP\left(R^{3}+3R_{0}^{3}h\right)$$

*Q* is the heat production, *μ* is the coefficient of kinetic friction, *N* is the rotational speed, P is the downward pressure, *R* is the radius of the shaft shoulder, *R*_0_ is the radius of the stirring needle, and h is the length of the stirring needle. Heat production is directly proportional to the needle length; the longer the needle length, the greater the amount of heat produced [29,30].

### 3.3. Mechanical Properties of Joints

**Figure 6:**
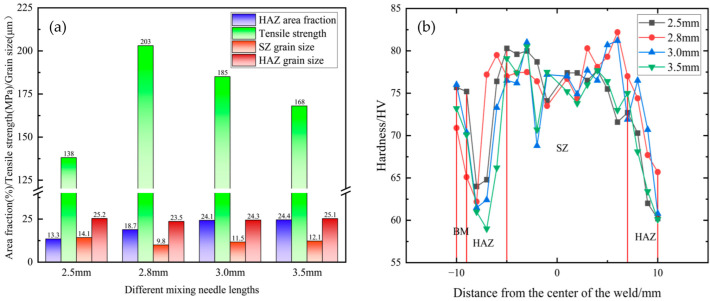
Mechanical properties of A380/6061 dissimilar aluminium alloys FSW joints with different stirring pin lengths: (**a**) mechanical properties and microstructural characteristics; (**b**) hardness distribution map.

Figure 6a shows the tensile strength and zone grain size graphs corresponding to four FSW joints made using different needle lengths increasing from 2.5 mm to 3.5 mm. It can be seen that the tensile strength first rises and then decreases, reaching a peak of 203 MPa at 2.8 mm. The grain size of its SZ zone is only 9.8 μm, which is smaller than that of the base material, and from the welding temperature of 2.8 mm, it can be seen that grain refinement occurs in the weld core zone [31], the RS-HAZ is tightly bonded with the weld core zone, and the weld core zone has great fluidity. This could be the main reason for its high tensile strength and great performance, and the area of the RS-HAZ accounts for 18.7% of the whole weld, in addition to the heat transfer output also being suitable. In contrast, at 2.5 mm, the tensile strength only reached a minimum of 138 MPa, at which time the area of the HAZ was only 13.3%, and the grain size of the SZ zone was 14.1 μm. This was because the pin length was too short, the heat generated during stirring was too low, and the stirring of the material was only limited to the upper layer of the material being welded. The two materials did not mechanically bond well in the weld core zone, resulting in a large number of band defects. Further, the mechanical and microstructural properties of the material deteriorated. At a 3.0 mm needle length, the area of the HAZ was 24.1%, and the grain size of the weld core zone was 11.5 μm, exhibiting grain refinement. Although the weld core zone achieved great fluidity, there were still a large number of band defects in the RS-HAZ, which might be due to the high welding temperature and excessive heat input, resulting in the material not being able to cool in time [32]. At a 3.5 mm needle length, the welding temperature was higher, the area of the HAZ accounted for 24.4%, and the grain size of the weld core zone was 12.1 μm. Further, the heat input was too high, the heat output was larger, the material could not cool in time, and the weld core zone had poorer fluidity, which degraded the material’s microstructure and properties. Figure 6b shows the hardness distribution of the weld with different needle lengths; the overall hardness exhibits an ‘M’ type distribution, and the hardness of the HAZ is the lowest in the entire weld. This may be because of the thermoplasticity of the A380 base material, which causes grain regrowth in the RS-HAZ for all four different needle lengths [33]. The hardness of the material was consequently reduced and was uniformly distributed in the weld core zone.

**Figure 7:**
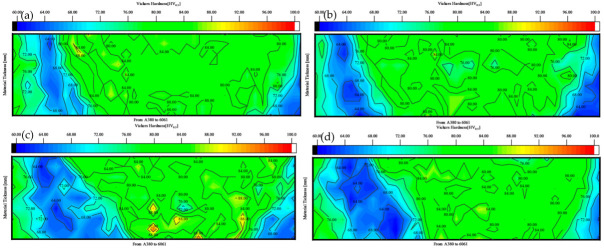
Hardness cloud maps at different mixing pin lengths for A380/6061 dissimilar aluminium alloys FSW joints: (**a**) 2.5; (**b**) 2.8; (**c**) 3.0; and (**d**) 3.5 mm.

**Figure 8:**
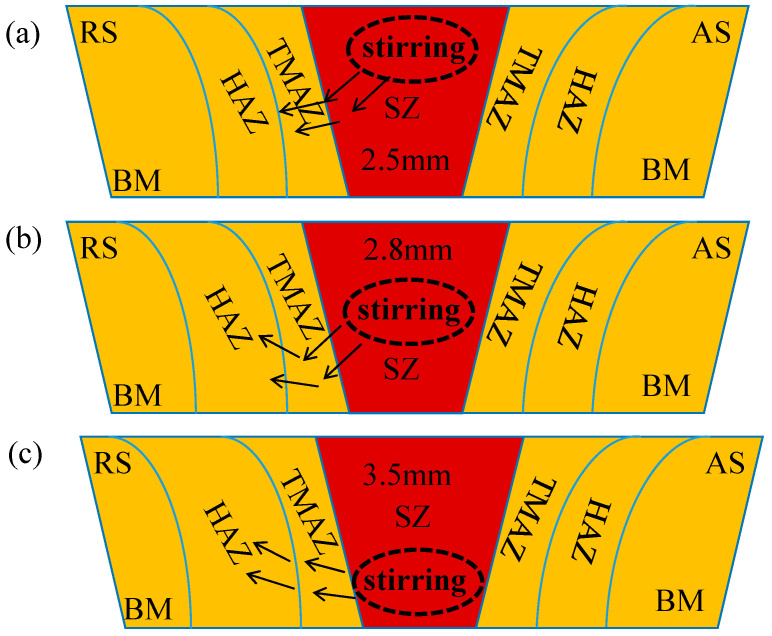
Schematic diagram of changes in RS-HAZs of A380/6061 dissimilar aluminium alloy FSW joints under thermal influence with different stirring pin lengths.: (**a**) 2.5; (**b**) 2.8; (**c**) 3.5mm.

Figure 7 shows the microhardness cloud at four different needle lengths. Figure 7a shows the hardness map at a 2.5 mm needle length; it can be seen that the area of the HAZ is relatively small, primarily because the stirring needle does not extend to the bottom, the heat generated is too low, the compound layer of the two materials is too thin, and the mechanical properties of the joints deteriorate. Figure 7b shows the microhardness cloud diagram at a 2.8 mm needle length, where the area of the HAZ increases, the stirring needle extends to the middle of the material, the heat generated is sufficient, and the two materials are significantly mechanically mixed in the weld core zone. The microstructural properties are very good. Figure 7c,d are microhardness cloud diagrams at 3.0 mm and 3.5 mm needle lengths, respectively; the area of the HAZ of the material continues to increase because the stirring needle extends to the bottom of the material, where it stirs vigorously. This generates excessive heat, such that the SZ zone microstructures cool very late [34], deteriorating the mechanical properties of the joints. The hardness of the HAZ is the lowest in the entire weld; the area increases, and the mechanical properties of the welded joint worsen. Figure 8 shows the RS-HAZ affected by heat change when stirring occurs in the upper layer, where the heat transfer is insufficient, the RS-HAZ area shrinks, and the weld core zone mobility is insufficient. When the position is in the middle, the heat transfer is more sufficient, the HAZ area increases, and the weld core zone mobility is great. When the position is at the bottom, the heat transfer is excessive, the RS-HAZ area is too large, and the weld core zone mobility becomes poor. The performance of the material also deteriorates.

### 3.4. Joint Microstructure

**Figure 9:**
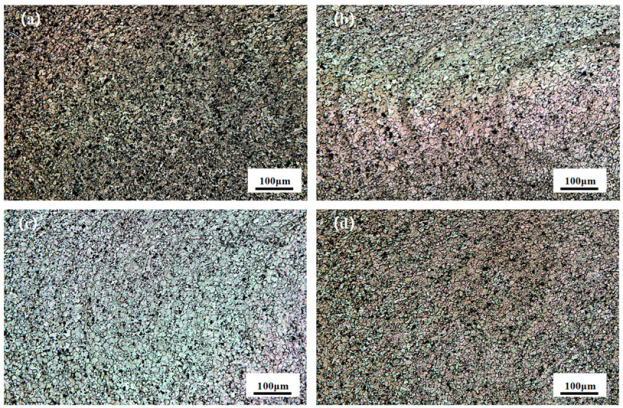
Microstructure of weld core area of A380/6061 dissimilar aluminium alloys FSW joints with different stirring pin lengths: (**a**) 2.5; (**b**) 2.8; (**c**) 3.0; and (**d**) 3.5 mm.

Figure 9 shows the microstructures and morphology of the welded nucleation zone of FSW joints under an optical microscope with four different needle lengths, as shown in Figure 9a. It is clear that the flowability of the nucleation zone under the needle length of 2.5 mm is poor, which is because the needle length of the FSW joint is too short, the heat generated is insufficient, and the two materials are not sufficiently stirred. As shown in Figure 9b, the flowability of the nuclear zone at a needle length of 2.8 mm is high, a layer-by-layer annular structure can be seen, and the heat generated during stirring is sufficient to form a strong mechanical bond between the two materials in the stirred zone. As shown in Figure 9c,d, the flow in the welded core zone is weakened under 3.0 and 3.5 mm needle lengths, which is due to the excessive heat generated; in addition, the stirring needles stir rapidly at the bottom of the material, which deteriorates the plastic flow of the material.

**Figure 10:**
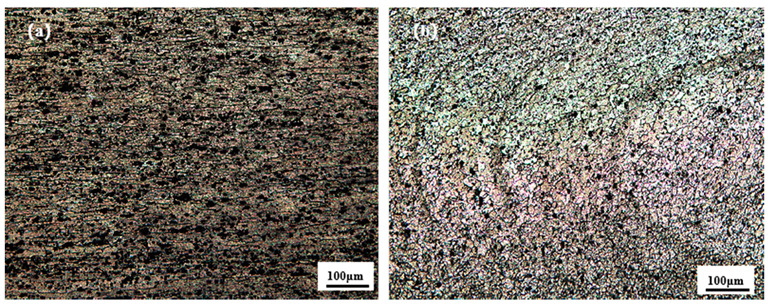
Microstructure of different areas of A380/6061 dissimilar aluminium alloy FSW joints with a 2.8 mm pin length: (**a**) HAZ; (**b**) weld core zone.

Figure 10 shows the microstructure of different regions at differing needle lengths. Figure 10a shows the microstructures of the RS-HAZ region after stirring, where the grains in this region are flattened with an average grain size of 25.7 μm, primarily due to the poor thermoplasticity of the A380 base material during stirring [35]; this results in grain regrowth. The grain size in this region is slightly larger than that of the A380 base material, and the hardness in this region is also the lowest where most of the tensile samples fracturing. Figure 10b shows the microstructures of the weld core region. The weld core area of the grain is granular, the average size of the grain is 9.8 μm, and the average size is smaller than the base material’s 6061-grain size. Because the stirring needle stirred strongly in this area, a large amount of heat was generated, which led to dynamic recrystallisation and grain refinement; further, mobility is obvious in the weld core area, indicating that the material has good mechanical properties.

**Figure 11:**
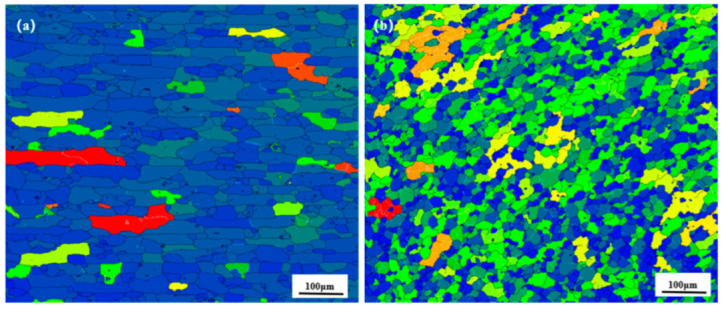
Weld grain orientation map of A380/6061 dissimilar aluminium alloys FSW joints with 2.8 mm pin lengths: (**a**) RS-HAZ zone; (**b**) weld core zone.

**Figure 12:**
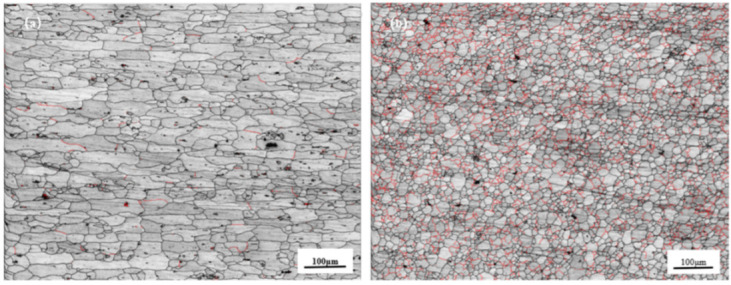
Weld grain distribution of A380/6061 dissimilar aluminium alloys FSW joints with 2.8 mm pin lengths: (**a**) RS-HAZ zone; (**b**) weld core.

**Figure 13:**
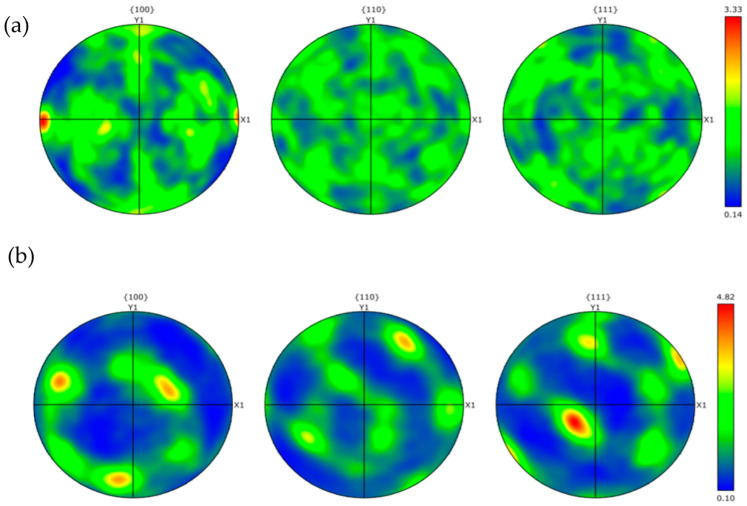
EBSD pole for different regions of A380/6061 dissimilar aluminium alloys FSW joints with 2.8 mm pin lengths: (**a**): RS-HAZ region; (**b**): welded core region.

Figure 11 and Figure 12 show the distribution of grain boundaries and the distribution of grain orientation in different regions of 2.8 mm-pin-length joints, respectively, and Figure 13 shows the grain orientation pole diagram. The blue line represents the high-angle grain boundary (greater than 15°), and the green line represents the low-angle grain boundary (2–15°). In the RS-HAZ, because of the lack of thermoplasticity of the BM A380 material, there are far more high-angle grain boundaries than low-angle grain boundaries. The proportion of high-angle grain boundaries is 95.4%, and the proportion of low-angle grain boundaries is 4.6%. Although the thermoplasticity of A380 aluminium alloy is insufficient, its grain size increases significantly after the mechanical action of the FSW joint. The average size of the grain increases by about 4.6 μm, and the number of low-angle (2–15°) grain boundaries in the weld core area is greater than the number of high-angle grain boundaries (greater than 15°), with low-angle grain boundaries accounting for 31% of the total and high-angle grain boundaries accounting for 69%. The 6061 aluminium alloy has great thermoplasticity, and due to the mechanical stirring and a large amount of heat input, the material undergoes significant plastic deformation in the weld core area, and the high-angle grain boundaries are transformed into low-angle grain boundaries, leading to dynamic recombination [36]. The grain size was refined and reduced by 5.9 μm compared to the 6061 base material. Figure 13 shows the pole diagrams of grain orientation in different regions of the welded joints with a 2.8 mm pin length, where it can be seen that the weaving strength of the welded core region is significantly weaker in comparison to the RS-HAZ region at this pin length.

**Figure 14:**
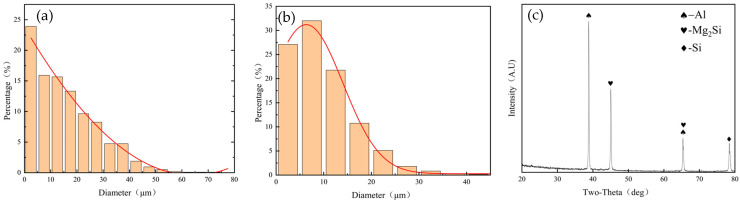
Histogram of grain size frequency distribution in different regions of A380/6061 dissimilar aluminium alloys FSW joints with 2.8 mm pin lengths: (**a**) RS-HAZ zone; (**b**) welded core zone; and (**c**) XRD spectrum of the weld core region of A380/6061 dissimilar aluminium alloys FSW joints with 2.8 mm pin lengths.

Figure 14a shows the RS-HAZ grain size sub-frequency distribution, mainly for small-sized grains; however, large-sized grains accounted for a relatively high proportion of the weld core zone. Figure 14b shows the weld core zone grain size frequency distribution, with sizes of 10 μm or less accounting for 59.7%, indicating that the weld core zone has good microstructural properties with fine grains.

Figure 14c shows the XRD analysis of the weld core region at the weld interface under the 2.8 mm pin length. It can be seen that a separate Al phase, Si phase, and Mg_2_Si were detected at the weld interface. This indicates that a Mg–Si metal compound layer was formed at the weld, which means that the two materials were bonded metallurgically, with good material properties. No separate Mg elements were detected, which is probably because of the low content of Mg elements that mostly formed Mg–Si metal compounds.

### 3.5. Fracture Discussion

**Figure 15:**
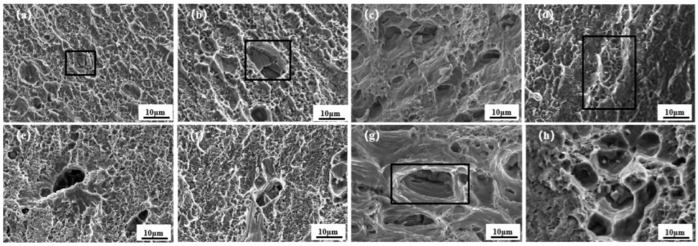
Tensile fracture morphology of A380/6061 dissimilar aluminium alloys FSW joints with 2.8 mm pin lengths: (**a**,**e**) 2.5; (**b**,**f**) 2.8; (**c**,**g**) 3.0 and (**d**,**h**) 3.5 mm.

**Figure 16:**
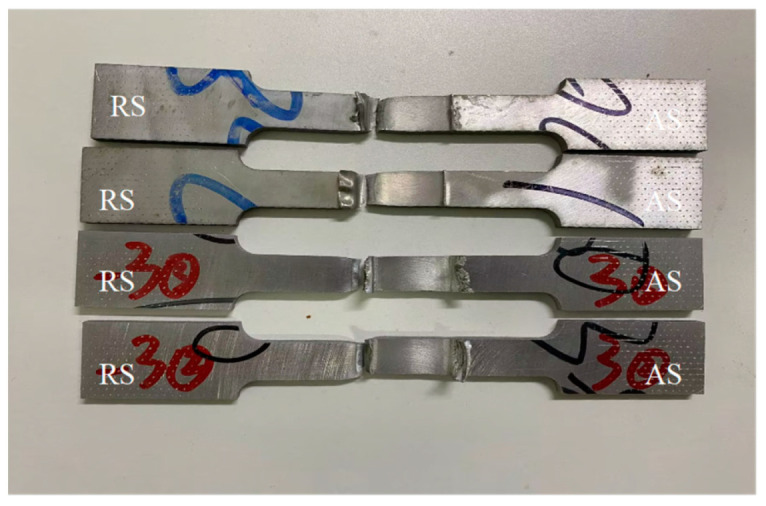
Schematic diagram of fracture of tensile specimen of A380/6061 dissimilar-alumini-um alloys FSW joints.

Figure 15 shows the fracture SEM morphology of the tensile specimens corresponding to four different stirring needle lengths. Figure 15a–h represent the morphology of the tensile fracture under four different needle lengths, respectively. In Figure 15a,b,e,f there are a large number of tough nests on the fracture surface; they reflect brittle fracture, which is a typical characteristic of the fracture of microporous polymerisation. Further, due to the location of the fracture in the HAZ region on the backward push side, the grains in this region are coarse, and the mechanical properties are weak. Obvious horseshoe-shaped defects also appear in Figure 15, and the distribution of tough nest holes is not uniform; it can be understood to be a mixed fracture. Figure 15c,d,g,h, appear similar to the honeycomb-like morphology, with the presence of a large number of different sizes of ligamentous fossae; the larger ligamentous fossae have some reinforcement-phase particles. In the presence of the tearing prongs, there is plastic deformation of the reinforcement, which indicates toughness fracture. Further, tensile fracture at different pin lengths is shown to be a mixed fracture. Figure 16 shows the post-fracture morphology of the tensile specimen, and it can be seen that the fracture location is in the RS-HAZ, which coincides with the abovementioned hardness cloud maps display.

## 4. Conclusions

1. When the spindle speed is 1000 rpm, and the welding speed is 120 mm/min, a 2.8 mm needle length for welding can obtain great fluidity of the weld, with the highest tensile strength. The joint strength coefficient is 85.3%, and the weld core area has a lot of Mg_2_Si. The average grain size in the weld core area is 9.8 μm, and the fracture mode of the tensile specimens is mixed fracture.
2. Under the mechanical action of the stirring needle, grain refinement occurs in the weld core zone, with a large number of low-angle grain boundaries; grain regrowth occurs in the RS-HAZ, with a large number of high-angle grain boundaries. The hardness of the RS-HAZ is also the lowest, and most of the tensile specimens are fractured here. There is no significant difference in the hardness values of the RS-HAZ at different needle lengths.
3. Under the same welding parameters, the length of the stirring needle affects the plastic flow at the bottom. When the length of the stirring needle is increased, the stirring area at the bottom of the material increases, resulting in poorer fluidity in the weld core area. When the stirring needle length decreases, the stirring needle cannot reach the bottom, and the heat generated is insufficient, which also makes the flowability of the weld core area worse. Therefore, a reasonable range of stirring needle lengths has a significant influence on the welding performance of the A380/6061 FSW joints.

## Figures and Tables

**Table 1:** Chemical composition of A380B and 6061 (mass fraction, wt%).

Alloys	Fe	Cr	Mn	Zn	Ti	Si	Cu	Sn	Mg	Al
A380	2.00	-	0.50	3.00	-	8.00	3.00	0.35	0.10	Bal
6061	0.20	0.05	0.10	0.10	0.10	0.40	0.10	-	0.40	Bal

## Data Availability

The original contributions presented in the study are included in the article, further inquiries can be directed to the corresponding author.
